# Quantitative framework for ordered degradation of APC/C substrates

**DOI:** 10.1186/s12915-015-0205-6

**Published:** 2015-11-16

**Authors:** Dan Lu, Juliet R. Girard, Weihan Li, Arda Mizrak, David O. Morgan

**Affiliations:** Departments of Physiology and Biochemistry & Biophysics, University of California, San Francisco, CA 94158 USA

**Keywords:** Cell cycle, Anaphase, APC/C, Cdc20, Model, Clb5, Securin, Ubiquitin

## Abstract

**Background:**

During cell-cycle progression, substrates of a single master regulatory enzyme can be modified in a specific order. Here, we used experimental and computational approaches to dissect the quantitative mechanisms underlying the ordered degradation of the substrates of the ubiquitin ligase APC/C^Cdc20^, a key regulator of chromosome segregation in mitosis.

**Results:**

We show experimentally that the rate of catalysis varies with different substrates of APC/C^Cdc20^. Using a computational model based on multi-step ubiquitination, we then show how changes in the interaction between a single substrate and APC/C^Cdc20^ can alter the timing of degradation onset relative to APC/C^Cdc20^ activation, while ensuring a fast degradation rate. Degradation timing and dynamics depend on substrate affinity for the enzyme as well as the catalytic rate at which the substrate is modified. When two substrates share the same pool of APC/C^Cdc20^, their relative enzyme affinities and rates of catalysis influence the partitioning of APC/C^Cdc20^ among substrates, resulting in substrate competition. Depending on how APC/C^Cdc20^ is partitioned among its substrates, competition can have minor or major effects on the degradation of certain substrates. We show experimentally that increased expression of the early APC/C^Cdc20^ substrate Clb5 does not delay the degradation of the later substrate securin, arguing against a role for competition with Clb5 in establishing securin degradation timing.

**Conclusions:**

The degradation timing of APC/C^Cdc20^ substrates depends on the multi-step nature of ubiquitination, differences in substrate-APC/C^Cdc20^ interactions, and competition among substrates. Our studies provide a conceptual framework for understanding how ordered modification can be established among substrates of the same regulatory enzyme, and facilitate our understanding of how precise temporal control is achieved by a small number of master regulators to ensure a successful cell division cycle.

**Electronic supplementary material:**

The online version of this article (doi:10.1186/s12915-015-0205-6) contains supplementary material, which is available to authorized users.

## Background

Progression through the cell cycle is accompanied by dramatic changes in cellular content and behavior, and involves a large number of proteins and processes. These changes are orchestrated by a small number of master regulators, including the cyclin-dependent kinases (Cdks) and the anaphase-promoting complex/cyclosome (APC/C). Each Cdk or APC/C isoform has a large number of substrates, and the substrates of each isoform are modified in a specific order that leads to sequential substrate activation or inactivation. This ordering of substrate modification allows a small number of master regulators to carry out their functions over a large time window with high temporal resolution, enabling precise and robust control of the numerous processes underlying cell cycle progression [1, 2].

The APC/C is an E3 ubiquitin ligase that drives mitotic progression by promoting timely degradation of key regulatory proteins [3]. In early mitosis, the APC/C associates with its activator subunit Cdc20 and promotes degradation of the S cyclin, cyclin A (in mammals) or Clb5 (in yeast), followed by securin several minutes later [4–6]. Securin degradation then unleashes separase to promote sister-chromatid separation. APC/C^Cdc20^ also initiates the degradation of M cyclin, cyclin B (in mammals) or Clb2 (in yeast), around the same time as securin [5, 6]. The activator Cdc20 is then replaced by a second activator, Cdh1, and APC/C^Cdh1^ promotes complete degradation of M cyclin, followed by polo-like kinase 1, Aurora A, and other substrates, to complete mitosis and cytokinesis and drive progression into G1 [1, 2].

The APC/C, as a multi-subunit RING-domain E3 ubiquitin ligase, acts as a platform that brings together a substrate and an E2 charged with ubiquitin, thereby catalyzing ubiquitin transfer from the E2 to the substrate (Additional file 1: Figure S1A) [3, 7, 8]. Specific substrates generally bind with sufficient affinity that they remain bound during multiple ubiquitin transfer reactions, resulting in processive substrate modification [9, 10]. Ubiquitins can be added to multiple lysine residues on the substrate, or can be added to pre-attached ubiquitins to build a polyubiquitin chain. These chains are recognized by the proteasome, leading to substrate degradation [11].

The activator Cdc20 or Cdh1 recruits substrate to the APC/C by interacting with short linear sequence motifs or degrons, such as the D box and KEN box [3, 8, 12]. In addition to recruiting substrates, the activator subunit induces a conformational change in the APC/C core, thereby enhancing E2 binding and the rate of ubiquitin transfer [13, 14]. Interestingly, the D box and KEN box of substrates contribute to this activation process. When substrates are directly fused to the APC/C core, thus bypassing the need of the D box and the KEN box for the recruitment of substrates to the APC/C, the presence of these degron motifs still increases the ability of the activator to promote ubiquitin transfer [14]. Thus, substrate degrons could potentially influence both the affinity for the APC/C and the catalytic rate of ubiquitin transfer.

APC/C^Cdc20^ activation depends on the expression of Cdc20 and phosphorylation of core APC/C subunits [15, 16]. Complete APC/C^Cdc20^ activation also requires inactivation of the spindle assembly checkpoint (SAC), which monitors proper attachment of sister chromatids to the spindle [17, 18]. In mammalian cells, cyclin A is degraded when the SAC is on, while securin degradation begins when the SAC is turned off, resulting in their sequential degradation [4, 19]. In the budding yeast *Saccharomyces cerevisiae*, we recently showed that the SAC is turned off earlier than securin degradation, and the different timing of Clb5 and securin degradation is due to several other mechanisms (Additional file 1: Figure S1B-E) [6]. In addition to its canonical D box, yeast Clb5 has an ABBA motif that mediates an extra interaction with Cdc20. Clb5 also interacts with Cdk1 in a complex with the phosphate-binding accessory subunit, Cks1. Cdk1-Cks1 binds directly to phosphorylated APC/C core subunits [15] and can thus bridge Clb5 to APC/C. These mechanisms make Clb5 an excellent APC/C^Cdc20^ substrate, and Clb5 is the first substrate to be degraded when APC/C^Cdc20^ becomes active [6]. Similar mechanisms promote cyclin A degradation in the presence of an active SAC in mammalian cells [20–22]. Yeast securin degradation, on the other hand, is delayed by Cdk1 phosphorylation near its D box and KEN box, and lags behind Clb5. When all these differences are eliminated, the degradation of Clb5 and securin occurs around the same time [6].

In our previous single-cell studies, differences in the degradation timing of different substrates (calculated as the time when 50 % of the substrate is degraded) were accompanied by relatively minor differences in their rates of degradation. For example, Clb5 is degraded six minutes earlier than securin, while the difference in their apparent half-life is less than 1.5 min (Additional file 1: Figure S1F) [6]. Thus, the overall timing of substrate degradation may be determined primarily by the timing of degradation onset. If true, this leads to an interesting question: if the mechanisms mentioned above make one substrate better than another, how do these mechanisms change the timing of degradation onset while having a relatively small effect on the rate of degradation? In other words, why don’t later substrates begin to be degraded as soon as APC/C^Cdc20^ becomes active?

In the current work, we show that differences in degradation onset indeed account for a major part of the overall differences in degradation timing for Clb5 and securin, and we explore how this differential onset is achieved. We first show that the ABBA motif of Clb5 increases the catalytic rate of ubiquitin transfer when Clb5 is bound to APC/C^Cdc20^, confirming that different substrates can be modified at different rates. We then build computational models that capture the interaction between the substrate and APC/C^Cdc20^, and we use these models to test the effects of substrate-specific changes in binding affinity and catalytic rate. In a one-substrate system lacking competition among different substrates, a multi-step ubiquitination process can produce a robust delay in substrate degradation relative to APC/C^Cdc20^ activation, while maintaining a rapid degradation rate like that we observed in the cell. In a two-substrate system with a limited amount of APC/C^Cdc20^, competition can further influence the timing of degradation by limiting the amount of APC/C available for each substrate. The degradation timing of substrates is thus determined collectively by substrate-APC/C^Cdc20^ interactions and APC/C^Cdc20^ partitioning among substrates, both of which are influenced by differences in kinetic parameters among substrates. We then show experimentally that increasing the amount of Clb5 does not significantly delay the degradation of securin, arguing that competition with Clb5 is not a major determinant of securin degradation timing in the cell. Finally, we use a modified one-substrate model to show that processivity in substrate ubiquitination is related to but not entirely predictive of degradation timing.

## Results

### Substrates of APC/C^Cdc20^ have different times of degradation onset

In our previous work, we established a single-cell assay to track the degradation dynamics of APC/C^Cdc20^ substrates in the budding yeast *Saccharomyces cerevisiae*. We tagged each substrate with green fluorescent protein (GFP) to follow its change in concentration over time. In the same strain, we also tagged the spindle-pole body (SPB) component Spc42 with mCherry. The SPB is seen as one dot in G1 and S phase, and separates into two dots at the onset of mitosis. At the metaphase-anaphase transition, the spindle begins to elongate and the two SPB dots move quickly away from each other. These two SPB events serve as single-cell timing references that allow us to align and compare GFP-tagged substrate degradation dynamics in different cells, either from the same strain or with different GFP-tagged substrates [6].

Previously, we estimated degradation timing by using the time point when 50 % of the GFP-tagged substrate was degraded. Here, we re-processed our previously published data to determine the timing of degradation onset. For each single-cell GFP trace, we smoothed and normalized the curve and calculated its first derivative (Additional file 1: Figure S2A). The minimum of the smoothed first derivative curve corresponds to the fastest declining point on the GFP trace during substrate degradation (Additional file 1: Figure S2A; yellow dots). Working backwards in time from that point, we identified the latest time point at which the smoothed first derivative was close to zero, which we used as our estimate of the time of degradation onset (Additional file 1: Figure S2A, B; green dots).

For all the substrates we analyzed, differences in substrate degradation timing were accompanied by significant differences in the timing of degradation onset (Additional file 1: Figure S3). Most relevant for this work are three substrates: Clb5, securin-2A, and Clb5-2A. The degradation onset of securin-2A, in which the inhibitory Cdk phosphorylation sites are mutated (T27A, S71A), begins an average of four min after Clb5 (Additional file 1: Figure S3A, Student's *t*-test, *p*-value <0.001). We focused on securin-2A instead of securin because these Cdk phosphorylation sites establish a positive feedback loop in securin degradation that could complicate our computational modeling [23]. Clb5-2A, in which the ABBA motif is mutated (I102A and Y103A), begins to be degraded three min later than wild-type Clb5 (Additional file 1: Figure S3B, Student's *t*-test, *p*-value <0.001). Since the ABBA motif allows Clb5 to compete with spindle assembly checkpoint (SAC) proteins for Cdc20 binding [6, 20], it is possible that the delay in Clb5-2A degradation relative to wild-type Clb5 is due to weak remaining SAC activity that does not inhibit Clb5 degradation. However, when we disable the SAC by deleting its key effector protein Mad2, Clb5-2A degradation is delayed relative to Clb5 to the same extent as it is in wild-type cells (Additional file 1: Fig S3C, D). Therefore, we attribute the delay in Clb5-2A degradation to a change in the Clb5-2A-APC/C^Cdc20^ interaction that is independent of the SAC.

The onset of Clb5 degradation indicates the time at which the SAC is turned off and APC/C^Cdc20^ first becomes active [6]. Thus, other substrates, such as Clb5-2A and securin-2A, are degraded several minutes after APC/C^Cdc20^ becomes active. It is worth noting that in the strain where Clb5-2A degradation was monitored, wild-type Clb5 is deleted and there are no known APC/C^Cdc20^ substrates that are better than Clb5-2A (Additional file 1: Figure S1C-E). Nevertheless, Clb5-2A degradation is delayed even though the timing of APC/C^Cdc20^ activation should remain the same.

### Clb5 ABBA motif increases the catalytic rate of Clb5 ubiquitination

**Fig. 1:**
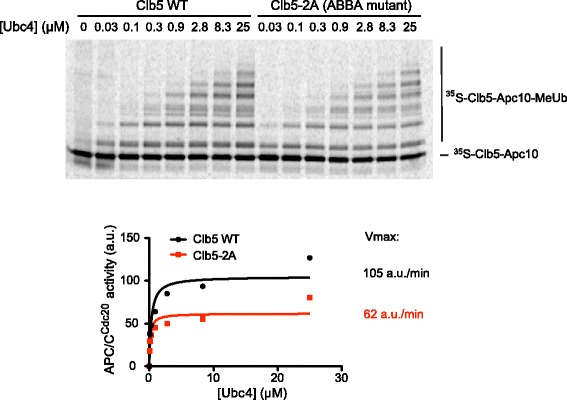
The ABBA motif of Clb5 increases the catalytic rate of ubiquitin transfer. APC/C^Cdc20^ activity was measured with a radiolabeled Clb5-Apc10 fusion protein [14] containing the N-terminal 150 residues of Clb5, either wild-type or carrying the 2A mutations in the ABBA motif. Reactions were performed in the presence of increasing concentrations of E2, and reaction products were subjected to SDS-PAGE and autoradiography with a PhosphorImager. The total amount of ubiquitinated substrate was quantified with ImageQuant software and plotted to determine maximal activity (a.u., arbitrary units). Results are representative of three independent experiments. *APC/C* anaphase-promoting complex/cyclosome

Our next goal was to develop computational models to help us understand how differences among substrates translate into distinct times of degradation onset. First, we needed to assess the parameters that might vary among substrates. The ABBA motif of Clb5 (or cyclin A in mammals) interacts with Cdc20, and for cyclin A this interaction is known to increase the binding affinity for Cdc20 [6, 20]. We tested an additional possibility that is based on our previous evidence that the D box and KEN box of securin increase the rate of ubiquitination once securin is bound to APC/C^Cdc20^ [14]. We wondered whether the ABBA motif could also increase the catalytic rate of Clb5 ubiquitination. We carried out APC/C^Cdc20^ ubiquitination reactions *in vitro* to directly measure the catalytic rate of Clb5 ubiquitination with or without the ABBA motif. We used a modified APC/C reaction in which the substrate is directly fused to the APC/C core subunit Apc10/Doc1 [14]. In this system, the enzyme is essentially saturated with substrate, and therefore differences in substrate affinity do not have an impact on the reaction rate. Some ubiquitination occurs in the absence of activator subunit, but addition of activator (Cdc20 or Cdh1) greatly enhances activity by improving the efficiency of the interaction with the E2 [14]. We fused the N-terminal 150 residues of Clb5 (containing the D box and the ABBA motif) to Apc10, generated radiolabeled fusion protein by translation *in vitro*, and incubated the fusion protein with purified APC/C^Cdc20^ lacking the Apc10 subunit. As in our previous work [14], we carried out activity measurements over a broad range of E2 concentrations. Mutation of the ABBA motif caused a reproducible 1.5- to 2-fold decrease in maximal catalytic activity (Fig. 1). Thus, APC/C substrates can differ not only in their binding affinities for the APC/C, but also in their catalytic rate of ubiquitin transfer.

### A simple dynamic model for APC/C^Cdc20^-mediated substrate ubiquitination and degradation

**Fig. 2:**
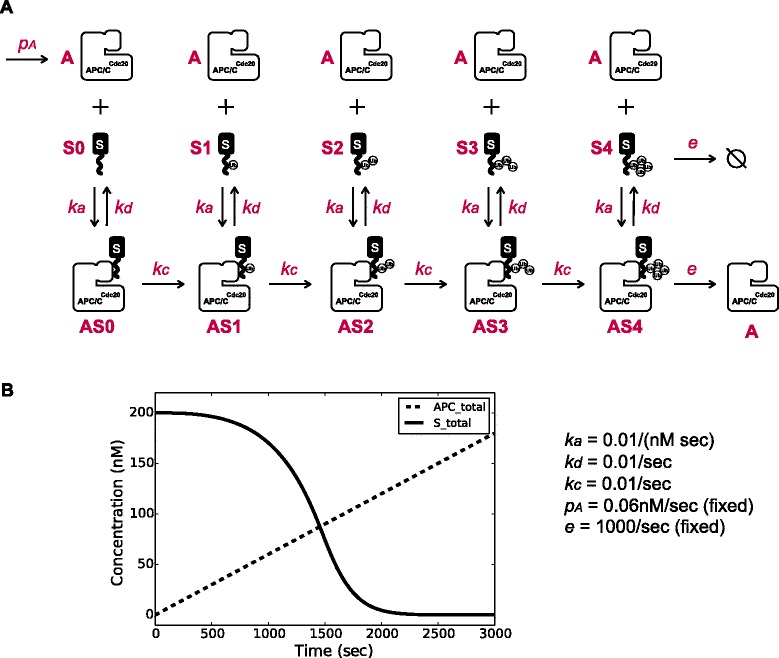
A dynamic model for substrate degradation. **a** The components of the model. A: free APC/C^Cdc20^, S: free substrate, AS: APC/C^Cdc20^-substrate complex, *k*_*a*_: association rate constant, *k*_*d*_: dissociation rate constant, *k*_*c*_: catalytic rate constant, *e*: degradation rate constant, *p*_*A*_: accumulation rate of active APC/C^Cdc20^. See Additional file 1: Figure S4 for equations. **b** An example of a degradation profile of a substrate at one parameter set. *APC/C* anaphase-promoting complex/cyclosome

We developed a computational model to determine whether it is possible, in principle, to generate a robust delay in substrate degradation by simply relying on the interaction between one substrate and APC/C^Cdc20^ (Fig. 2a). The model includes the following molecular species: free APC/C^Cdc20^ (A); free unmodified substrate (S0), free substrate with one, two, three or four ubiquitins attached (S1, S2, S3, S4, respectively); APC/C^Cdc20^-bound unmodified substrate (AS0), and APC/C^Cdc20^-bound substrate with one, two, three or four ubiquitins attached (AS1, AS2, AS3, AS4, respectively). These molecular species interact and interconvert by the following rate constants: APC/C^Cdc20^ and free substrate associate with the rate constant *k*_*a*_; APC/C^Cdc20^-bound substrates can either dissociate with rate constant *k*_*d*_ or can be modified by the attachment of ubiquitin with the rate constant *k*_*c*_*.* Once a substrate carries four ubiquitins, regardless of whether it is bound to APC/C^Cdc20^ or not, it is degraded by the proteasome with rate constant *e*. The amount of active APC/C^Cdc20^ increases linearly at rate *p*_A_ starting from zero APC/C^Cdc20^ at the zero time point. Substrate starts at a fixed amount and we assume no production of substrate, since our previous work showed that the level of Clb5-2A plateaus for several minutes before its degradation begins (Additional file 1: Figure S3B, D)[6]. The concentration change of each molecular species was determined by ordinary differential equations (Additional file 1: Figure S4), and all reactions were modeled as mass action since we considered binding and catalysis steps explicitly.

The initial concentration of the free substrate was set at 200 nM, based on previous estimates of Clb5 concentration in the cell [24]. We fixed the rate of APC/C^Cdc20^ accumulation *p*_*A*_ at 0.06 nM/sec, based on estimates of Cdc20 concentration obtained by single-cell analysis of GFP-tagged Cdc20 (Additional file 1: Figure S5). The degradation rate constant *e* was fixed at 1,000/sec, ensuring that substrate was degraded as soon as it was modified with four ubiquitins. All other parameters were varied across a range of values. We varied the substrate ubiquitination and dissociation rate constants *k*_*c*_ and *k*_*d*_ from 10^−3^/sec to 10^3^/sec, based in part on previous enzymatic reaction results *in vitro* [9, 14, 25]. Substrate association rate constant *k*_*a*_ varied from 10^−4^/(nM sec) to 10/(nM sec) (that is, 10^5^ to 10^10^/[M sec]). Note that *k*_*a*_ should be similar for different substrates as it is mostly determined by rates of random collisions, but we still analyzed a range of *k*_*a*_ values to gain insights about the system.

We evaluated our model at 25 values for *k*_*c*_ and *k*_*d*_ and six values for *k*_*a*_, all evenly distributed on a log scale, so that each *k*_*c*_ and *k*_*d*_ value was changed by a factor of 1.8, and *k*_*a*_ changed by a factor of 10, as compared to its immediate neighbors. For each set of parameters, we calculated the dynamics of substrate concentration over a period of 50 min, which is similar to the duration of a movie for experimental analysis (Fig. 2b).

### Delay in degradation onset and fast degradation rate are opposing constraints

**Fig. 3:**
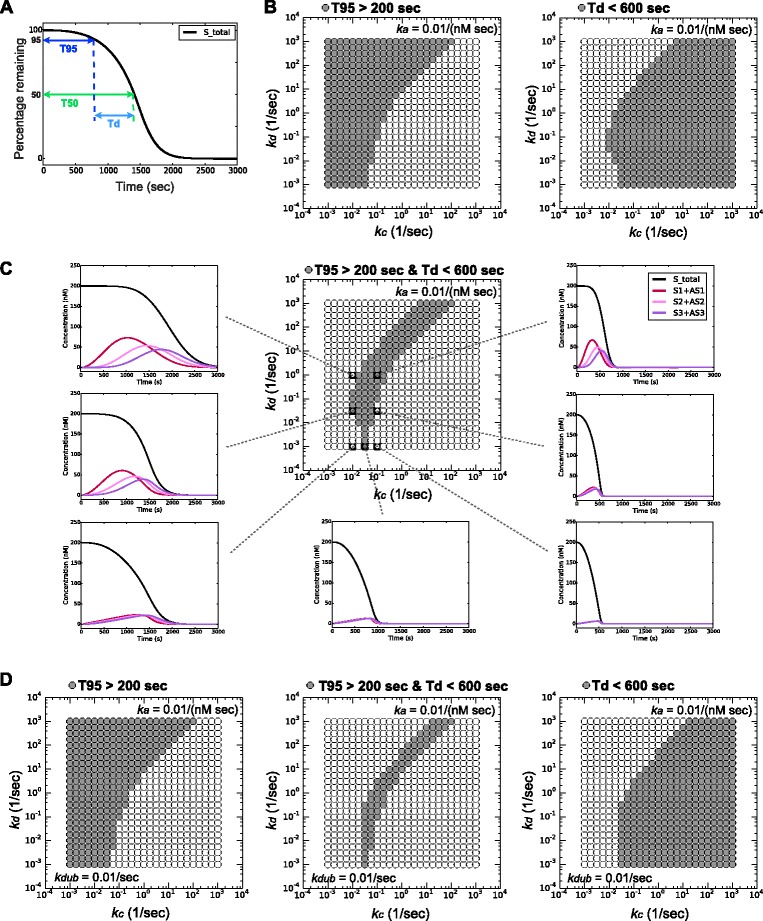
A delay in degradation onset and a fast rate of degradation are opposing constraints. **a** Measurement of T95 as an estimate of the delay in degradation onset, and Td as an estimate of degradation rate. **b** The *k*_*c*_-*k*_*d*_ parameter regions where T95 > 200 sec or Td < 600 sec, as indicated by grey dots. *k*_*a*_ = 0.01/(nM sec). **c** *Center*: the parameter region where T95 > 200 sec and Td < 600 sec, as indicated by grey dots. *Surrounding plots*: substrate degradation profiles with different parameter combinations as indicated by the dotted lines. Black curves show the total amount of substrate; red, pink, and purple curves show the intermediate products with one, two or three ubiquitins attached, respectively. *k*_*a*_ = 0.01/(nM sec). **d** Incorporation of a deubiquitination reaction (with rate constant *k*_*dub*_) for all ubiquitinated substrates slightly expands the parameter region with a good delay (T95 > 200 sec) but reduces the region with a fast rate (Td < 600 sec)

Using the one-substrate APC/C^Cdc20^ model, we first searched for the parameter space that generated two behaviors seen in our previous *in vivo* studies: a significant delay in degradation onset and a rapid degradation rate. For each set of parameters, we quantified the delay in degradation onset by measuring the duration from time zero (i.e., the onset of APC/C^Cdc20^ activity) to the time when substrate concentration declined to 95 % of its initial value (T95). To estimate the rate of substrate degradation, we measured the duration from T95 to the time when 50 % of the substrate was degraded (Td = T50 - T95) (Fig. 3a). Based on our experimental data, we were looking for the set of model parameters that had a T95 greater than 200 sec as well as a Td of less than 600 sec. It was immediately clear that these two criteria are satisfied by opposing constraints. At any fixed substrate association rate constant *k*_*a*_, a long T95 requires a small ubiquitination rate constant *k*_*c*_ and/or a large substrate dissociation rate constant *k*_*d*_, whereas a short Td requires a large *k*_*c*_ and/or a small *k*_*d*_ (Fig. 3b). Therefore, the parameter space where both criteria are satisfied is restricted to a small overlap zone. Within this zone, we can easily reproduce Clb5-2A or securin-2A degradation dynamics like those observed *in vivo* (Fig. 3c).

### Deubiquitination helps establish a delay at a cost to degradation rate

The ubiquitination state of a protein is determined by the relative rates of ubiquitination and deubiquitination. We thus analyzed the role of deubiquitination in our model by incorporating a deubiquitination reaction for all substrates with ubiquitins attached, bound to APC/C^Cdc20^ or not. This led to an increase in the delay of degradation onset but also reduced the degradation rate, and was not essential to reproduce the experimentally observed delayed substrate degradation onset (Fig. 3d). We also tried allowing deubiquitination only for free substrates, only for APC/C^Cdc20^-bound substrates, or only for substrates with one ubiquitin attached [26], and obtained similar results (Additional file 1: Figure S6).

### Varying the dissociation rate constant *k*_*d*_ influences degradation timing when free APC/C^Cdc20^ is available

**Fig. 4:**
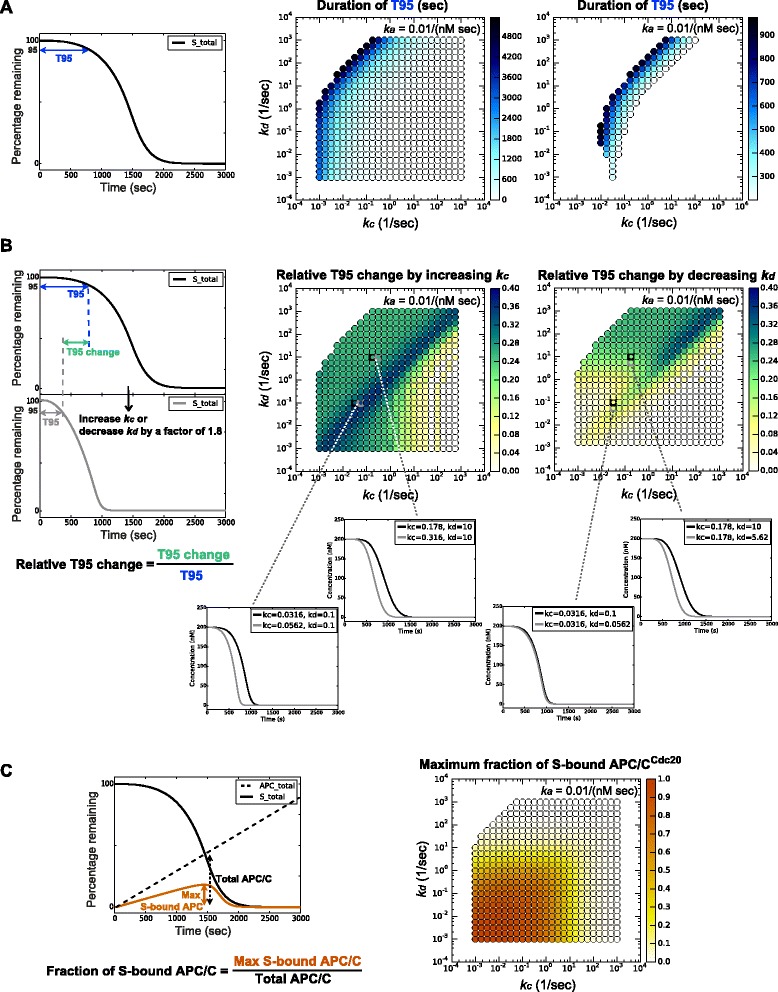
Varying *k*_*c*_ and *k*_*d*_ influences T95 differently depending on amount of free APC/C. Each dot represents one pair of (*k*_*c*_ *,k*_*d*_) values, and the color corresponds to the quantity of interest shown on the example profiles on the *left*. In all panels, *k*_*a*_ = 0.01/(nM sec). **a** *Middle panel*: dot color indicates the duration of T95 (in seconds) at different *k*_*c*_ *-k*_*d*_ combinations. *Right panel*: a close-up of the parameter region that meets both T95 and Td requirements (as in Fig. 3c center panel), with an adjusted color scale. **b** *Middle panel*: at each *k*_*c*_ *-k*_*d*_ combination, dot color indicates the relative decrease of T95 when *k*_*c*_ was increased by a factor of 1.8. *Below* are substrate degradation profiles showing the effect of increasing *k*_*c*_ by a factor of 1.8 at the parameter combinations indicated by the dotted lines. *Right panel*: dot color indicates the relative decrease of T95 when *k*_*d*_ was decreased by a factor of 1.8. *Below* are substrate degradation profiles showing the effect of decreasing *k*_*d*_ by a factor of 1.8 at the parameter combinations indicated by the dotted lines. **c** Dot color indicates the maximum fraction of APC/C^Cdc20^ that is bound to S during the degradation process. See Additional file 1: Figure S7 for plots at different values of the association rate constant *k*_*a*_ *. APC/C* anaphase-promoting complex/cyclosome

To address how the timing of degradation might be changed for different substrates, we quantified the delay in degradation onset (T95) for substrates with different *k*_*c*_ and *k*_*d*_*.* We fixed *k*_*a*_ at 0.01/(nM sec) and calculated T95 for each combination of *k*_*c*_*-k*_*d*_ (Fig. 4a). As expected, increasing *k*_*c*_ (rightward along x axis) or decreasing *k*_*d*_ (downward along y axis) generally accelerated the ubiquitination process and decreased T95.

To measure the change in T95 that results from a small change in *k*_*c*_, we calculated the relative decrease in T95 following an increase in *k*_*c*_ by a factor of 1.8 (Fig. 4b, left panel), starting with every *k*_*c*_-*k*_*d*_ combination (Fig. 4b, middle panel). Increasing *k*_*c*_ decreased T95 significantly throughout the parameter space, except in the region where degradation occurs extremely fast due to large *k*_*c*_*.*

Similarly, we calculated the relative decrease in T95 after a decrease in *k*_*d*_ by a factor of 1.8 (Fig. 4b, right panel). Decreasing *k*_*d*_ caused a significant decrease in T95 primarily in the area where free APC/C^Cdc20^ is available (Fig. 4c; see also Additional file 1: Figure S7). In the lower left parameter region where varying *k*_*d*_ does not significantly influence T95, all APC/C^Cdc20^ molecules are occupied by substrate, ubiquitination occurs at the maximum rate, and varying *k*_*d*_ does not significantly change the amount of substrate-bound APC/C^Cdc20^.

### A model system with two substrates sharing the same pool of APC/C^Cdc20^ readily generates differences in degradation onset

Since the amount of Cdc20 is not in large excess over substrates *in vivo* (Additional file 1: Figure S5), competition among substrates for APC/C^Cdc20^ is possible. To understand how competition might influence substrate degradation timing, we analyzed a model with two substrates, C and S, that are based on the yeast substrates Clb5 and securin-2A. The two substrates start at the same concentration and interact with the same pool of APC/C^Cdc20^. The only difference is that C is a better substrate than S, either by having: (1) a smaller dissociation rate constant *k*_*d,C*_ that is 1/10 the *k*_*d,S*_ of S; or (2) a larger catalytic rate constant *k*_*c,C*_ that is 10-fold greater than the *k*_*c,S*_ of S.

**Fig. 5:**
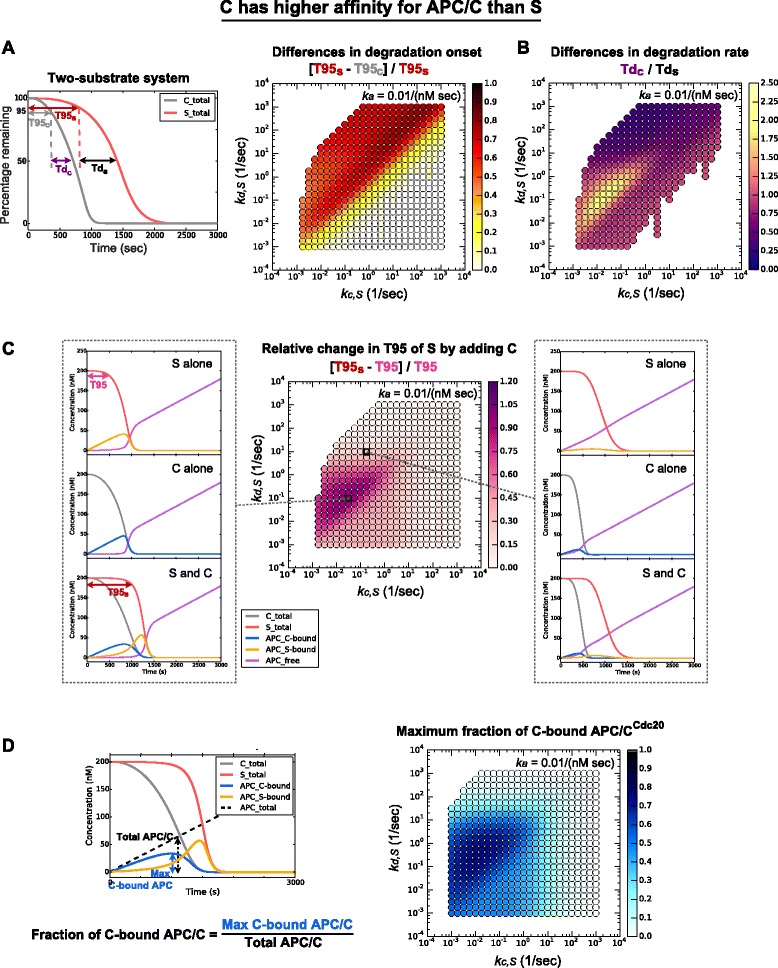
Two-substrate system in which C has higher affinity than S for APC/C^Cdc20^. The parameter space shown is *k*_*c*_ *-k*_*d*_ of S; at any parameter combination, *k*_*d,C*_ = 1/10*k_*d,S*_. **a** Relative difference in degradation onset (T95) comparing S to C. **b** Difference in degradation rates of C and S, shown as the ratio of Td of C over Td of S. **c** *Middle panel*: change of T95 of S in two-substrate system compared to one-substrate system. *Left* and *right panels*: degradation profiles of a one-substrate system and two-substrate system showing the dynamics of different molecular species at the parameter values indicated on the *middle panel* by dashed lines. **d** In the two-substrate system, the maximum fraction of C-bound APC/C^Cdc20^. *APC/C* anaphase-promoting complex/cyclosome

**Fig. 6:**
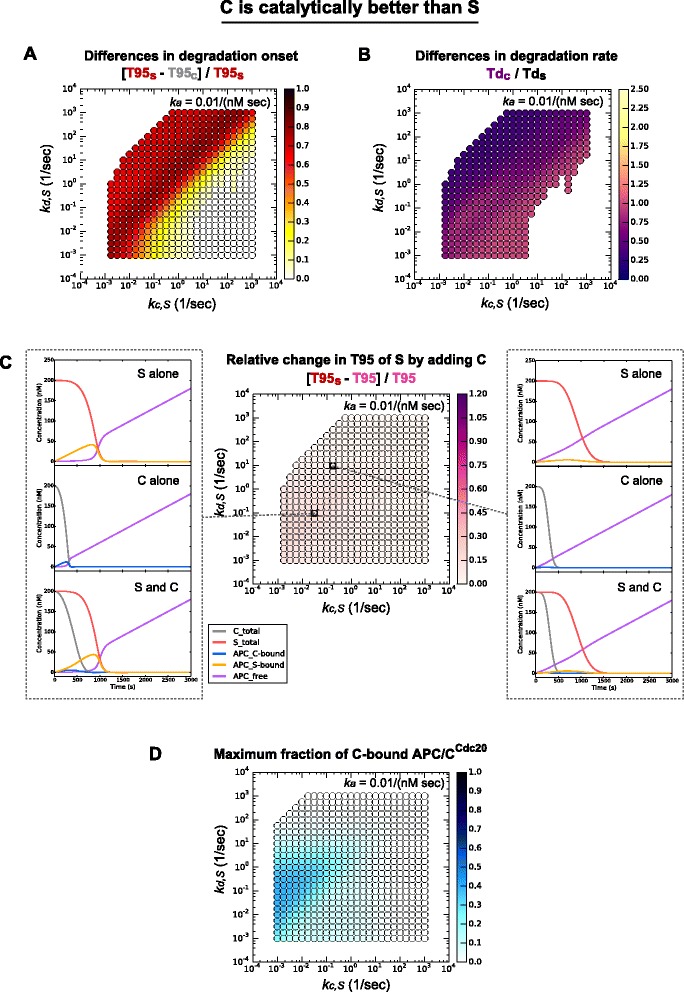
Two-substrate system in which C is catalytically better than S. The parameter space shown is *k*_*c*_ *-k*_*d*_ of S; at any parameter combination, *k*_*c,C*_ = 10*k_*c,S*_. **a** Relative difference in degradation onset (T95) comparing S to C. **b** Difference in degradation rates of C and S, shown as the ratio of Td of C over Td of S. **c** *Middle panel*: change of T95 of S in two-substrate system compared to one-substrate system. Left and right panels: degradation profiles of a one-substrate system and two-substrate system showing the dynamics of different molecular species at the parameter values indicated on the *middle panel* by dashed lines. **d** In the two-substrate system, the maximum fraction of C-bound APC/C^Cdc20^. *APC/C* anaphase-promoting complex/cyclosome

We first analyzed the differences in degradation onset (T95) between C and S in two-substrate systems. Regardless of the way in which C is a better substrate, we found a large parameter region in which there was a robust, significant difference in the timing of their degradation onset (red regions in Figs. 5a and 6a). The region where the difference in T95 was small had either: (1) a small *k*_*d,S*_, such that the difference between *k*_*d,S*_ and 1/10 *k*_*d,C*_ was too small to distinguish C and S and the two substrates were degraded with similar timing; or (2) a large *k*_*c*_, such that the degradation of both substrates was extremely fast (i.e., T95 was very small; white regions in Figs. 5a and 6a).

We then analyzed the differences in degradation rate (Td) between C and S (Figs. 5b and 6b). C generally had a higher degradation rate than S (Td_C_/Td_S_ < 1), whether its affinity for APC/C^Cdc20^ was higher (Fig. 5b) or its rate of catalysis was higher (Fig. 6b), which is consistent with our previous observation that the rate of Clb5 degradation *in vivo* is slightly higher than that of securin-2A (Additional file 1: Figure S1F) [6]. Only one parameter region did not generate this result: when C had higher affinity for APC/C^Cdc20^, the degradation rate of C was slower than that of S at some low values of *k*_*d,S*_ and *k*_*c,S*_ (Fig. 5b; yellow and orange region, Td_C_/Td_S_ > 1).

### Substrate competition could delay degradation by decreasing available APC/C^Cdc20^

We next explored in more detail how two different substrates influence each other’s degradation timing and rate, by comparing degradation of S in a two-substrate system to that in a one-substrate system with the same parameter values. We reasoned that in parameter regions where C has little effect on S degradation, the timing and rate of S degradation are determined primarily by its interaction with APC/C^Cdc20^ as in a one-substrate system, and all the properties of a one-substrate system apply.

We first analyzed the two-substrate system in which C binds APC/C^Cdc20^ with ten-fold higher affinity than S (*k*_*d,C*_ is 1/10 *k*_*d,S*_), as described above. We calculated the relative increase in the T95 of S following the addition of C to the system, compared to S being the only substrate. In one parameter region, the addition of C significantly delayed the degradation of S (Fig. 5c; left and middle panel, red region). Under these conditions, C competitively inhibits S degradation by occupying a large amount of APC/C^Cdc20^, thereby reducing the amount of APC/C^Cdc20^ available to S (Fig. 5d). In the other parameter regions, *k*_*d,S*_ is sufficiently large to allow more free APC/C^Cdc20^ in the one-substrate system (see Fig. 4c), and the additional C in the system occupies only a small fraction of APC/C^Cdc20^. This helps buffer against the effect of adding C to the system, and C does not delay S degradation significantly (Fig. 5c; right panel).

Interestingly, C did not have a significant impact on S in our alternate two-substrate system in which C is 10-fold more efficiently ubiquitinated once bound to APC/C^Cdc20^. Addition of C to this system had very little effect on the degradation of S in the entire parameter region (Fig. 6c). In this scenario, when C is bound to APC/C^Cdc20^, the substrate either dissociates or is rapidly ubiquitinated and destroyed. This short life-time of the C-APC/C^Cdc20^ complex results in a very small population of C-bound APC/C^Cdc20^ (Fig. 6d). Thus, C does not sequester APC/C^Cdc20^ away from S and has little influence on S degradation, and the delayed degradation of S is primarily established by the parameters of its interaction with the APC/C^Cdc20^ as in the one-substrate system. Interestingly, in some parameter regions in this scenario, S is more efficient in occupying APC/C^Cdc20^ and can delay degradation of C (Fig. 6c; left panel).

The two-substrate systems we analyzed are both extreme cases. In reality, Clb5 could be better than securin-2A due to relatively small improvements in both *k*_*d*_ and *k*_*c*_. For instance, our biochemical studies showed that the ABBA motif of Clb5 can increase its *k*_*c*_ (Fig. 1), and others have shown that the ABBA motif of cyclin A can decrease its *k*_*d*_ [18]. Other APC/C-interacting motifs are also likely to have similar effects on both *k*_*d*_ and *k*_*c*_ [14]. These parameters influence both the substrate-APC/C^Cdc20^ interaction and the effect of competition for limited amounts of APC/C^Cdc20^. For the Clb5-2A mutant that lacks the ABBA motif, the delayed degradation onset compared to Clb5 might be a combined result of being a less efficient APC/C^Cdc20^ substrate and possibly less efficient in competing with other substrates for APC/C^Cdc20^ binding.

### Similar conclusions are reached in models with constant APC/C^Cdc20^ activity

**Fig. 7:**
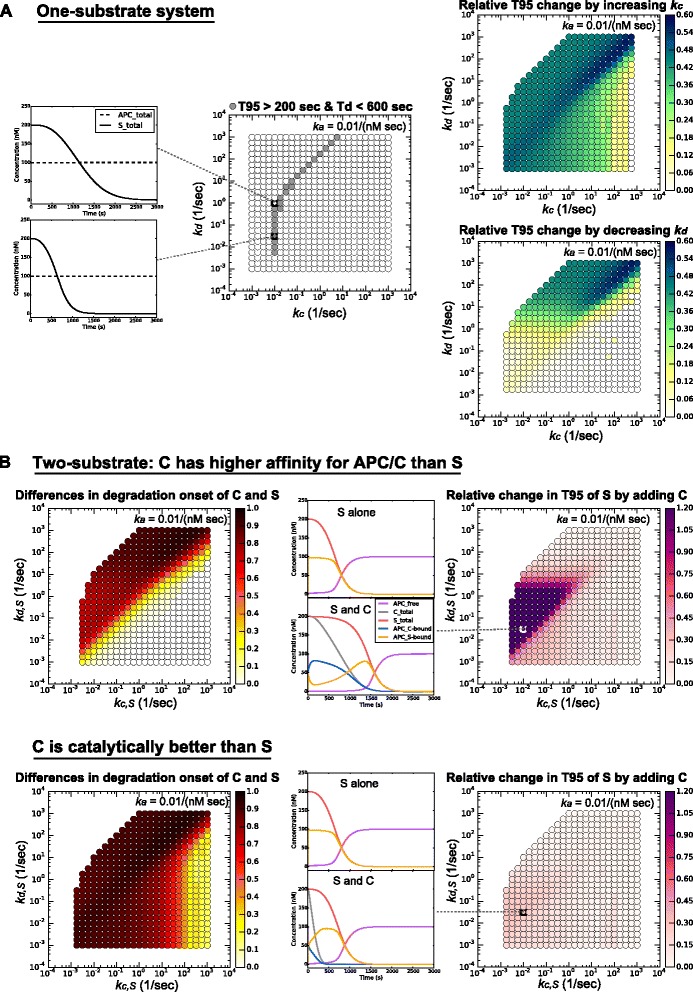
Alternative models with constant APC/C^Cdc20^ as input. **a** One-substrate system. These plots show the same quantities calculated in Figs. 3c and 4b, except using models in which APC/C^Cdc20^ activity increases abruptly and remains constant. **b** Two-substrate system. These plots show the same quantities calculated in Fig. 5a,c and Fig. 6a,c, except using models in which APC/C^Cdc20^ activity increases abruptly and remains constant. *APC/C* anaphase-promoting complex/cyclosome

In the modeling described thus far, we used linearly increasing APC/C^Cdc20^ activity as a simple approximation of the condition in the cell. To explore further the effects of different patterns of APC/C^Cdc20^ activation, we carried out computational studies of one- and two-substrate systems with a constant level of APC/C^Cdc20^ (100 nM, half the initial concentration of substrates), as might be the case when APC/C^Cdc20^ activity increases abruptly (perhaps due to SAC inactivation) to maximal levels. The major effect of constant APC/C^Cdc20^ activity is that the system has a smaller parameter region with both delayed degradation onset and rapid degradation rate (Fig. 7a). We explain this result as follows: larger amounts of APC/C^Cdc20^ activity exist at earlier time points in this system than in the system with gradually increasing APC/C^Cdc20^ activity, and this reduces the delay in degradation onset. At later time points, APC/C^Cdc20^ is not increasing and this reduces the degradation rate. Other than these changes, however, all other conclusions from our previous models remain unchanged: one substrate can still exhibit a robust delay in its degradation onset, and adding a second substrate may or may not increase this delay, depending on how APC/C^Cdc20^ is partitioned among the substrates (Fig. 7b).

### Clb5 does not significantly delay securin degradation

**Fig. 8:**
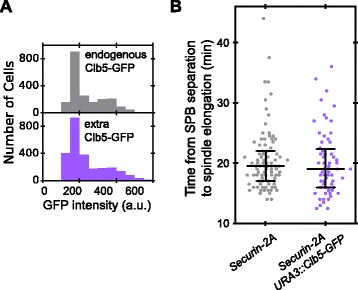
Extra Clb5 does not significantly delay securin-2A degradation *in vivo*. **a** A strain was constructed in which an extra copy of *CLB5*, tagged with GFP and under the control of its own promoter, was introduced into a *securin-2A* strain. The expression level of the extra copy of Clb5-GFP is similar to that in a different strain in which endogenous Clb5 was tagged with GFP. **b** Time from SPB separation (mitotic onset) to spindle elongation (anaphase onset) with one or two copies of Clb5 in the cell. *GFP* green fluorescent protein

If competition from Clb5 contributes significantly to the delay in securin-2A degradation, then the delay should be reduced by removing Clb5 and increased by adding more Clb5. Direct deletion of Clb5 causes DNA replication defects and slows cell-cycle progression, which would complicate our measurement of mitotic timing [27]. We therefore decided to add more Clb5 to the cell by introducing an extra copy of *CLB5*, driven by its own promoter, into cells with securin replaced by the securin-2A allele. These cells maintain their endogenous copy of Clb5. The extra copy of Clb5 was tagged with GFP to confirm its expression in the cell (Fig. 8a). The presence of the extra Clb5 did not delay spindle elongation relative to SPB separation (Fig. 8b). Since spindle elongation is directly driven by securin-2A degradation, we conclude that the timing of securin-2A degradation was unaffected by extra Clb5. These results are most consistent with a two-substrate model in which Clb5 has a higher *k*_*c*_, does not occupy a significant fraction of the APC/C^Cdc20^, and therefore does not compete effectively with securin *in vivo* (Additional file 1: Figure S8).

### Processivity is determined by a subset of the factors that determine degradation timing and dynamics

**Fig. 9:**
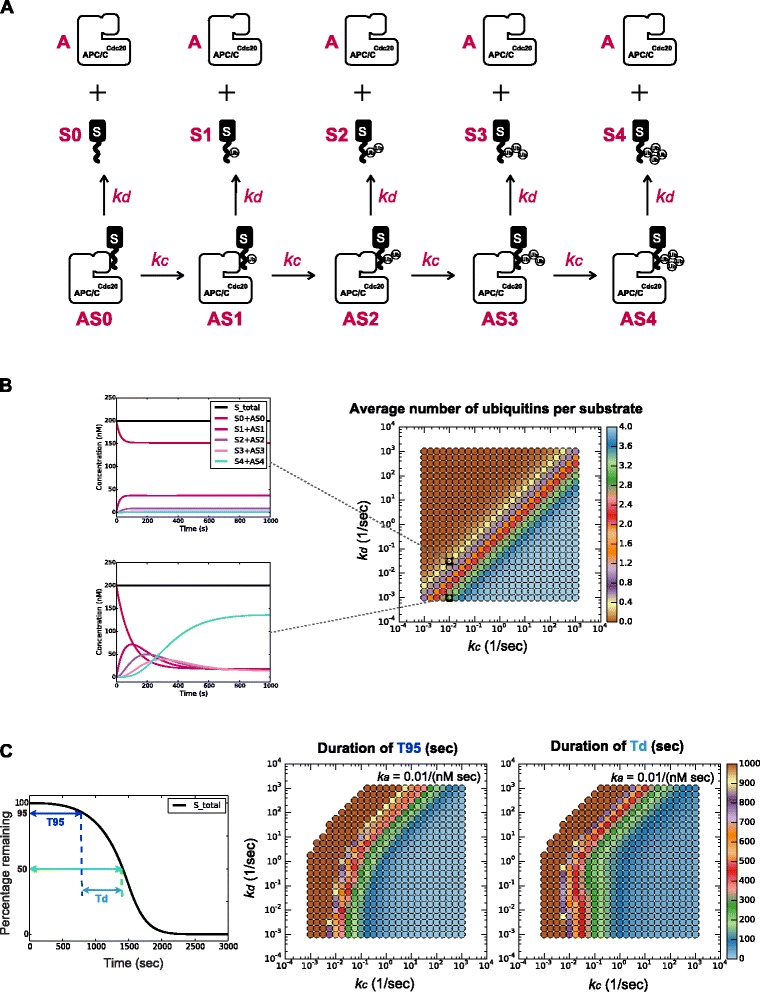
The relationship between processivity and degradation timing or dynamics. **a** A modified one-substrate model to simulate processivity. The system starts with AS0 as the only component. Each AS complex can either obtain one ubiquitin or dissociate into A and S. **b** Graphs at *left* show the amounts of each species as the system approaches steady state over time. The plot on the *right* shows the average number of ubiquitins per substrate, calculated as follows: when all substrates have dissociated from APC/C, no more reactions occur and the system reaches steady state, the total number of ubiquitins attached to all substrates is divided by the total number of substrates. **c** T95 and Td were calculated with the one-substrate system illustrated in Fig. 2. The T95 plot shows the same data as in Fig. 4a, color-coded to provide more detail and to facilitate comparison with panel **b** above. *APC/C* anaphase-promoting complex/cyclosome

The APC/C is processive: more than one ubiquitin can be attached during a single substrate-binding event (i.e., when the catalytic rate exceeds the substrate dissociation rate) [9]. Differences in processivity with different substrates are thought to influence the order of substrate degradation timing [10]. We explored this issue by analyzing the relationship between processivity and substrate degradation timing and dynamics with a modified one-substrate model. Processivity is measured as the number of ubiquitins attached to a substrate before it dissociates from APC/C^Cdc20^. Thus, a simple model to calculate processivity can start with S-bound APC/C^Cdc20^ (AS0) as the sole molecular species and does not require an association rate constant, in which case the concentration of free substrate or APC/C^Cdc20^ becomes unimportant (Fig. 9a). In the same *k*_*c*_-*k*_*d*_ parameter space that we analyzed in our other studies, we calculated the average number of ubiquitins per substrate molecule after the system reached steady state for each parameter combination. As expected, processivity, as a steady state property, is determined by the relative strength of *k*_*c*_ and *k*_*d*_ (Fig. 9b). However, higher processivity did not always correlate with earlier or faster degradation (Fig. 9c). We believe this is the case because T95 and Td, as dynamic properties, are determined not only by the absolute strength of *k*_*c*_ and *k*_*d*_ but also by additional factors, including the association rate constant *k*_*a*_ and the concentrations of free S and APC/C^Cdc20^. Thus, processivity is related to substrate degradation timing and dynamics but is not the sole determinant.

## Discussion

A key feature of the APC/C^Cdc20^ system is the multi-step ubiquitination process. A substrate that takes longer to acquire enough ubiquitins for efficient degradation, either due to lower affinity binding to APC/C^Cdc20^ or a slower rate of ubiquitination once it is bound, will take longer to initiate degradation. If two substrates differ only in their catalytic rate of ubiquitin transfer, they will have different timing of degradation onset (in both one- and two-substrate systems). If two substrates differ only in their binding affinity for APC/C^Cdc20^, then each substrate by itself (in a one-substrate system) may not have dramatically different degradation onset timing. However, when the two substrates share the same pool of APC/C^Cdc20^, the substrate with tighter binding affinity might sequester APC/C^Cdc20^ away from the other substrate to delay its degradation. As a result, regardless of whether substrates differ in their *k*_*c*_ or *k*_*d*_, they are very likely to have different times of degradation onset. Ordered substrate degradation is therefore likely to be the default output of the APC/C ubiquitination system.

It is important to emphasize that competition among substrates is not necessary for delayed substrate degradation relative to APC/C^Cdc20^ activation. The impact of competition depends on how APC/C^Cdc20^ is partitioned among its substrates, which is determined by the relative concentrations of APC/C^Cdc20^ and all of its substrates, and the relative strengths of substrate-specific kinetic parameters. These parameters are not readily determined inside the cell, and so the effect of competition *in vivo* is difficult to predict. Nevertheless, it is possible to gain some insight by testing the effects of small perturbations in substrate concentrations within a physiologically relevant range. We showed, for example, that an extra copy of Clb5 has no effect on the timing of securin degradation, suggesting that competition from Clb5 does not delay securin degradation. However, Clb5 and securin are not the only substrates in the cell that compete for APC/C^Cdc20^ binding. A complete understanding of competition in this system will require more extensive experiments that take all substrates into account.

Previous work unveiled multiple mechanisms that influence substrate degradation timing, such as the ABBA motif and Cdk1/Cks1 binding of Clb5, as well as the phosphorylation of securin. However, it is not clear which parameters these mechanisms actually influence. An obvious and very likely possibility is that many of these mechanisms simply change the affinity of a substrate for APC/C^Cdc20^, which can have an impact on the timing of degradation. Our experimental and modeling work also point to a less intuitive possibility: changes in substrate binding to APC/C^Cdc20^ can affect catalytic rate. Here, we showed that the ABBA motif increases *k*_*c*_ for Clb5 by nearly two-fold. Similarly, in previous work, we showed that binding of the D box and KEN box of substrates to the activator subunit helps promote catalytic function [14]. These results raise the interesting possibility that APC/C^Cdc20^ substrate binding and catalytic stimulation are intrinsically coupled, such that degrons enhance both affinity and catalysis. One potential explanation is that binding of additional degron motifs to the activator reduces the flexibility of disordered substrate regions flanking the binding sites, thereby reducing unproductive substrate orientations and allowing more efficient ubiquitin transfer. In addition, substrate-activator binding might help orient the activator to enhance the efficiency of E2 binding, and different substrates might be more or less effective in doing this.

A more complete understanding of the system requires knowledge of real rate constants *in vivo*, which are not well characterized. Catalytic rates measured in APC/C reactions *in vitro* are very low and seem unlikely to be reflective of real rates *in vivo*; for example, we recently observed that the catalytic rate constant of ubiquitin transfer with APC/C^Cdh1^ is roughly 0.001/sec with the E2 Ubc4, using a securin-Apc10 fusion protein as substrate [14]; this rate is likely to be lower than actual rates because it was measured with methylated ubiquitin. We are similarly uncertain about the affinity of substrates for the APC/C. The apparent K_M_ for sea urchin cyclin B with APC/C^Cdh1^ is 63 nM [9], and an equilibrium dissociation constant k_D_ in the range of 100 nM to 1 μM is a reasonable estimate for a substrate with two short linear degrons. If we assume that APC/C-substrate association is simply diffusion-limited, then the association rate constant *k*_*a*_ would be roughly 0.0001 ~ 0.001/(nM sec) (i.e., 10^5^ ~ 10^6^/(M sec)). If the k_D_ is 100 nM to 1 μM, then we might predict a dissociation rate constant *k*_*d*_ of 0.01 ~ 1/sec.

All of these values fall within the parameter space we studied. However, it is inappropriate to directly map these estimates on our parameter space, as the models greatly simplify the real system in several respects. For example, we assumed linearly increasing or constant APC/C^Cdc20^ levels, while APC/C^Cdc20^ activation dynamics *in vivo* could be much more complex. In addition, we considered only single ubiquitin chain formation, we did not explicitly model activator and E2 binding to APC/C^Cdc20^, and we assumed the same rate constants for all steps. It is also important to note that our experiments measure the degradation of substrate, which is a combined result of ubiquitination and proteasomal degradation. Since the mutants we analyzed specifically perturb the substrate-APC/C^Cdc20^ interaction, we focused on the ubiquitination process. It is possible, however, that substrate-specific differences in proteasomal degradation also contribute to the dynamics of substrate degradation.

By constructing simple models and scanning a broad range of reasonable parameters, our goal was not to reproduce precisely the behavior of substrates in the cell, but rather to obtain useful general insights into the key factors that determine the sequential modification of APC/C targets during progression through mitosis. Our conclusions are far from comprehensive and definitive, but we hope that they will motivate and serve as a primer for future investigations. It is likely that similar principles will help us understand the sequential phosphorylation and dephosphorylation of protein kinase targets during the cell cycle. Cdk1 substrates, for example, often have multiple phosphorylation sites, and ordered substrate phosphorylation is likely to depend on variations in docking motifs that confer differences in specificity (defined as *k*_*cat*_/K_M_) [28]. Similarly, the sequential dephosphorylation of yeast Cdk1 substrates in late mitosis is thought to be a combinatorial result of Cdk1 specificity and specificity of the phosphatase Cdc14 [29, 30]. Our work also emphasizes the connection and distinction between the two dynamic properties that are critical in the control of cell division: timing and rate, which we find are determined by the same sets of parameters but favor opposing trends of parameter values. Thus, for example, a deubiquitination reaction in our system facilitates the delay in degradation onset but also compromises the rate of degradation. These findings raise interesting questions about the strategies cells use to optimize both the timing and rate of cellular events, and how cellular circuits evolve under these constraints.

## Conclusions

Our goal in this work was to develop a quantitative framework to help us understand our experimental observations that: (1) different APC/C^Cdc20^ substrates begin to be degraded at different times; (2) some substrates do not start to decline until the APC/C^Cdc20^ has been active for several minutes; (3) once degradation begins, substrates are degraded within several minutes; and (4) earlier substrates degrade at a rate slightly faster than later substrates. A simple multi-step ubiquitination model is sufficient to recapitulate what we observe *in vivo*. Our modeling shows that delayed degradation onset and fast degradation rates are opposing constraints in the system. Our models also show that, at fixed substrate and APC/C^Cdc20^ concentrations, substrate degradation dynamics can be determined by a combination of substrate-APC/C^Cdc20^ interactions (as in the one-substrate model), and competition among substrates (as in the two-substrate model). Varying parameters, such as *k*_*c*_ and *k*_*d*_, influences both substrate-APC/C^Cdc20^ interactions and the competition among substrates, and thus results in changes in the onset and rate of degradation.

## Methods

### Yeast strain construction

All yeast strains were haploid derivatives of the W303 strain. Fluorescent protein tagging, gene replacement, and deletion of genes at their endogenous loci were performed using standard PCR-based homologous recombination [31–34], while preserving the endogenous promoters. Addition of Clb5-GFP to the genome was done using an integration plasmid at the genomic *URA3* locus [35], with its endogenous promoter, and selected for single-copy integration by PCR and fluorescence intensity.

### Fluorescence microscopy

All images were taken with a spinning-disk confocal microscope at the UCSF Nikon Imaging Center with a 60x/1.4 NA oil immersion objective, under the control of Micromanager [36]. The microscope is a Nikon Ti-E inverted microscope equipped with a Yokogawa CSU-22 scanner unit and a Photometrics Evolve EMCCD camera. Illumination was provided by a 50 mW 491 nm laser and a 50 mW 561 nm laser. Imaging sessions were generally 1 h long, with 30-sec time intervals. Z-stacks were taken across 4 μm of distance with 0.5 μm steps for each time point and each channel. Exposure times for mCherry and GFP channels were below 100 ms for each Z slice. All yeast cultures were grown and imaged at 30 °C. Prior to imaging, yeast cells were grown in synthetic complete media with 2 % glucose (SD) for 24 h with serial dilution to maintain OD below 0.4. For imaging, cells were mounted on a 1.5 % agarose pad made with SD media, and allowed to continue proliferating on the slide for 40–60 min in a 30 °C incubator prior to imaging.

### Image processing

To quantify GFP intensity at each time point, we first used ImageJ (http://imagej.nih.gov/ij/) [37] and its plugin Image5D (http://rsb.info.nih.gov/ij/plugins/image5d.html) to average across each z-stack and flatten it to 2D. GFP intensity was then quantified using MATLAB (The MathWorks Inc.) code previously developed in the Tang lab [38]. Since all of the APC/C substrates we studied were localized to the nucleus, we took the brightest square of 5×5 pixels in the cell as an estimate of the protein. Timing of SPB events was determined based on the temporal 3D positions of the SPB using the mCherry images [6]. SPB separation was defined as the time point when one SPB split into two, and spindle elongation was defined as the time point when two SPBs began to move rapidly away from each other.

The time point of substrate degradation onset in each cell was defined as when GFP intensity started to drop on a smoothed trace of a degradation event (Additional file 1: Figure S2). Determination of the time of degradation onset, or the level of GFP at a certain time point, was carried out with previously developed MATLAB code [6]. Statistical analysis and plotting were carried out in MATLAB and Python [39, 40].

### Western blotting

To measure levels of Cdc20, GFP-Cdc20, and Clb5-GFP (Additional file 1: Figure S5), log-phase cells were arrested in alpha-factor for 4 h at room temperature and released by washing. Samples were taken at various time points after release, and cells were lysed by bead-beating in lysis buffer (50 mM HEPES [pH 8.0], 150 mM NaCl, 1 % NP40, 50 mM beta-glycerophosphate, 50 mM NaF, 1 mM DTT, 1 μg/ml leupeptin, 1 μg/ml pepstatin, 1 μg/ml aprotinin, 1 mM PMSF, 10 % glycerol, 0.63 mg/ml benzamidine, and 5 mM EDTA). Lysates were analyzed by western blotting with anti-Cdc20 (yC-20/sc-6731, Santa Cruz Biotechnology, Santa Cruz, CA, USA).

### Ubiquitination assays in vitro

For analysis of APC/C activity with fusion substrates (Fig. 1) [14], Clb5-Apc10 fusion substrates were translated *in vitro* with TnT Quick Coupled Transcription/Translation Systems (Promega, Madison, WI, USA) in the presence of ^35^S-methionine. APC/C was purified from lysates of *CDC16-TAP cdh1∆ doc1∆* W303 strains by affinity chromatography with IgG beads [9]. E1 (Uba1-6His) was expressed in yeast and purified using ubiquitin agarose [9]. E2 (Ubc4-6His) was expressed in *E. coli* and purified by metal-affinity chromatography [41]. Cdc20 was tagged with an N-terminal ZZ tag and TEV cleavage site and produced with TnT Quick Coupled Transcription/Translation Systems (Promega, Madison, WI, USA), followed by purification on IgG beads and TEV cleavage of the ZZ tag [42]. E2 charging was performed in the presence of E1 (Uba1, 300 nM), E2 (Ubc4, 36 μM or varying concentrations), methyl-ubiquitin (Boston Biochem, Cambridge, MA, USA; 150 μM), and ATP (1 mM) in buffer containing 20 mM Hepes pH 7.4, 150 mM NaCl, 0.1 % Nonidet P-40, 1 mM MgCl_2_ at 23 °C for 20 min. Purified APC/C on IgG beads was incubated with fusion substrates at 23 °C for 30 min, and unbound substrate was washed away. Purified Cdc20 was then added, and reactions were initiated by mixing the Cdc20-APC/C-fusion substrate mixture with E2 charging mixture. After 20 min at 23 °C, reaction products were separated by SDS-PAGE and visualized with a Molecular Dynamics PhosphorImager. The data were analyzed using the software Prism (GraphPad).

### Computational modeling

Changes in the concentration of molecular species were determined by ordinary differential equations (Additional file 1: Figure S4). For each set of parameters, the simulation ran for 3,000 time steps corresponding to 3,000 seconds, with concentrations of every molecular species calculated at each time point. Using the dynamics of each substrate, we determined the quantity of interest, such as Td or T95. To cover a large parameter space, we carried out the simulation for 25 values of *k*_*c*_ and *k*_*d*_ ranging from 10^−3^/sec to 10^3^/sec equally spaced on a log scale, and six values of *k*_*a*_ ranging from 10^−4^/(nM sec) to 10/(nM sec). All dynamic simulations, data analysis, and plotting were carried out in Python with custom written code [39, 40, 43].

## Additional file

Additional file 1:
**Supplementary Figures. Figure S1.** Ordered degradation of APC/C substrates. **Figure S2.** Determination of the time of degradation onset. **Figure S3.** Mechanisms that determine substrate degradation timing all change degradation onset. **Figure S4.** Ordinary differential equations for analysis of substrate degradation in the one-substrate model. **Figure S5.** APC/C^Cdc20^ levels in the cell are lower than those of its substrates. **Figure S6.** Including deubiquitination in the model further limits the parameter space that generates a good delay in degradation onset and a fast rate of degradation. **Figure S7.** Changing *k*_d_ influences T95 when not all APC/C^Cdc20^ is bound to the substrate. **Figure S8.** Effects of doubling the concentration of C on T95 of S. (PDF 5283 kb)
