# Supplementary material for: Quantitative framework for ordered degradation of APC/C substrates
Source: BMC Biol. 2015 Nov 16;13:96. doi: 10.1186/s12915-015-0205-6 (PMC4647693; doi:10.1186/s12915-015-0205-6)
Supplement: Additional file 1: — Supplementary Figures. Figure S1. Ordered degradation of APC/C substrates. Figure S2. Determination of the time of degradation onset. Figure S3. Mechanisms that determine substrate degradation timing all change degradation onset. Figure S4. Ordinary differential equations for analysis of substrate degradation in the one-substrate model. Figure S5. APC/CCdc20 levels in the cell are lower than those of its substrates. Figure S6. Including deubiquitination in the model further limits the parameter space that generates a good delay in degradation onset and a fast rate of degradation. Figure S7. Changing k d influences T95 when not all APC/CCdc20 is bound to the substrate. Figure S8. Effects of doubling the concentration of C on T95 of S. (PDF 5283 kb) [file 12915_2015_205_MOESM1_ESM.pdf]

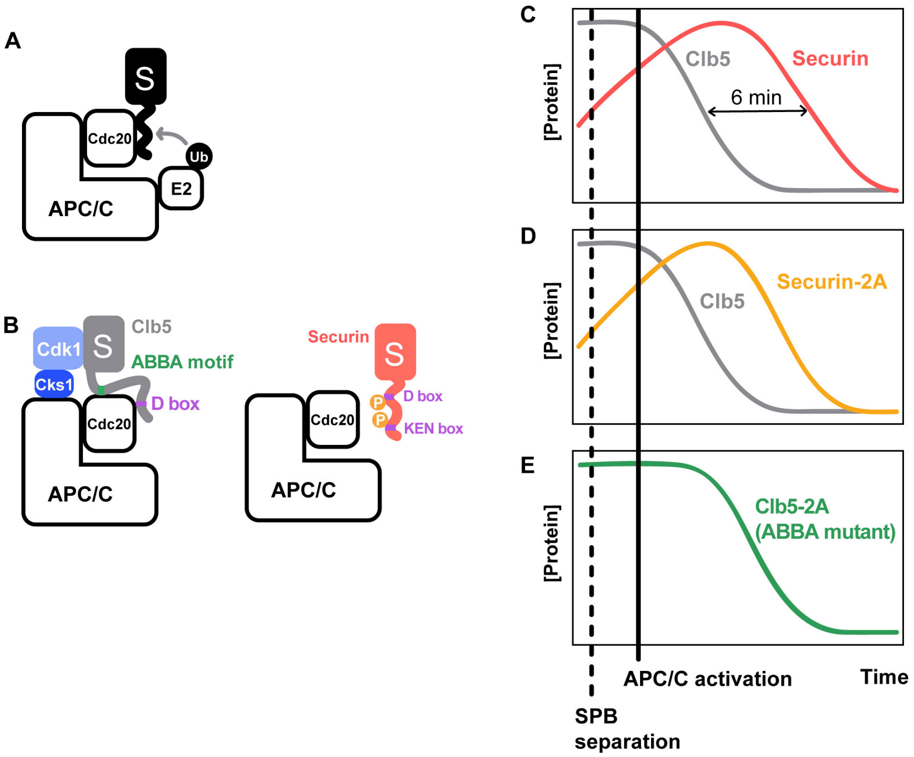

F

protein half-life (min)	25%	**median**	75%
Clb5	2.62	**3.25**	4.02
Clb5-2A	2.58	**3.28**	3.98
securin	3.15	**4.58**	5.92
securin-2A	3.09	**4.10**	4.85

Fig. S1 Ordered degradation of APC/C substrates. (A) APC/C^{Cdc20} brings E2-Ub and substrate together to promote ubiquitin transfer from E2 to the substrate. (B) Differences in the Clb5-APC/C^{Cdc20} interaction and securin-APC/C^{Cdc20} interaction determine the difference in their degradation timing. Clb5 has an extra degron, the ABBA motif, and interacts with the APC/C core indirectly through the Cdk1-Cks1 complex. Cdk1 phosphorylates securin near its degrons to delay securin destruction. (C-E) Degradation timing of different wild-type and mutant APC/C^{Cdc20} substrates. Dashed line indicates SPB separation, which serves as a timing reference to compare different GFP-tagged substrates *in vivo*. Solid line indicates APC/C^{Cdc20} activation, which is inferred from Clb5 degradation onset and will be time 0 in our modeling. Each panel represents one strain background. (C) Clb5 and securin. (D) Clb5 and securin-2A, which lacks the inhibitory phosphorylation sites. (E) Clb5-2A, which is mutated at the ABBA motif (securin in the same strain is omitted for clarity). Note the delay in Clb5-2A degradation onset relative to Clb5 in (C). (F) Degradation rates of different substrates are listed as protein half-lives, determined from previously published data (Lu D, Hsiao JY, Davey NE, Van Voorhis VA, Foster SA, Tang C, Morgan DO. J Cell Biol. 2014;207:23-39).

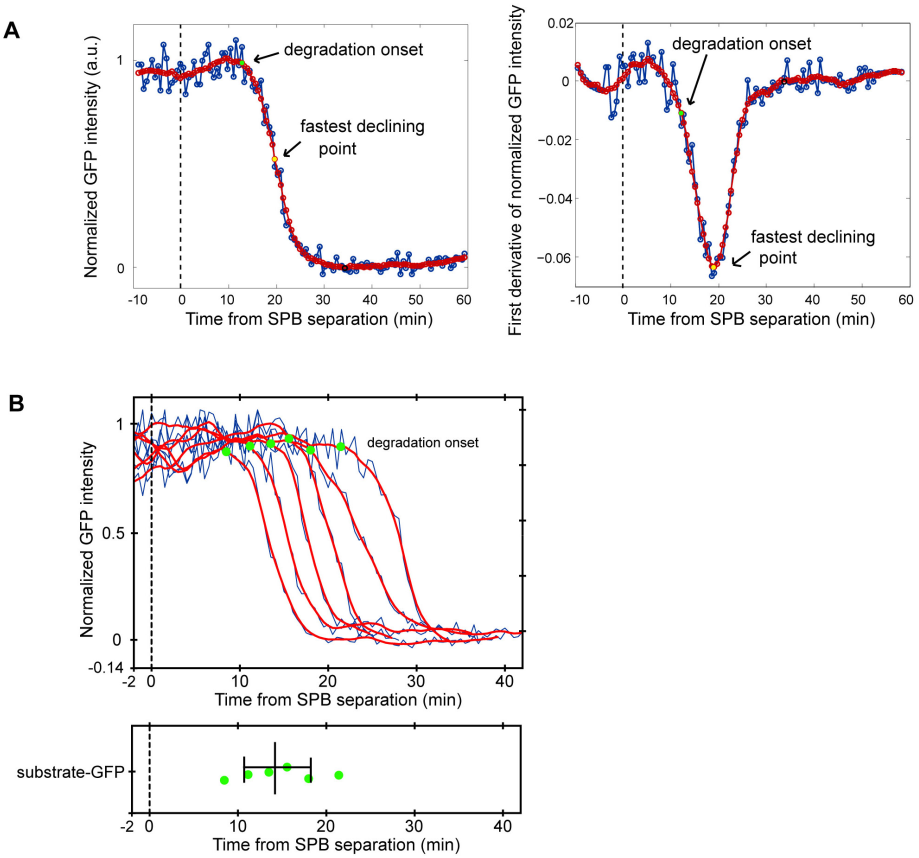

Fig. S2 Determination of the time of degradation onset. (A) Left panel: blue dots indicate the level of GFP-tagged substrate as a function of time, measured by fluorescence microscopy in a single cell, and the red curve is the smoothed GFP trace. The smoothed trace is then normalized to a range of 0 to 1. Right panel: blue dots indicate the first derivative of the normalized, smoothed GFP data, and the red curve is the smoothed first derivative. The fastest declining point of the GFP curve is the minimum of the first derivative (yellow dot). Starting from the time point of the yellow dot and going backward to where first derivative is close to zero (between 0 and -0.01) provides the time of degradation onset (green dot). (B) Plotting degradation onset timing of multiple single cells. Top panel: several single-cell GFP traces, with green dots to mark the time of degradation onset. Bottom panel: a plot of the time of degradation onset for the cells in the top panel; error bars indicate the 25th and 75th percentiles and the middle bar shows the median.

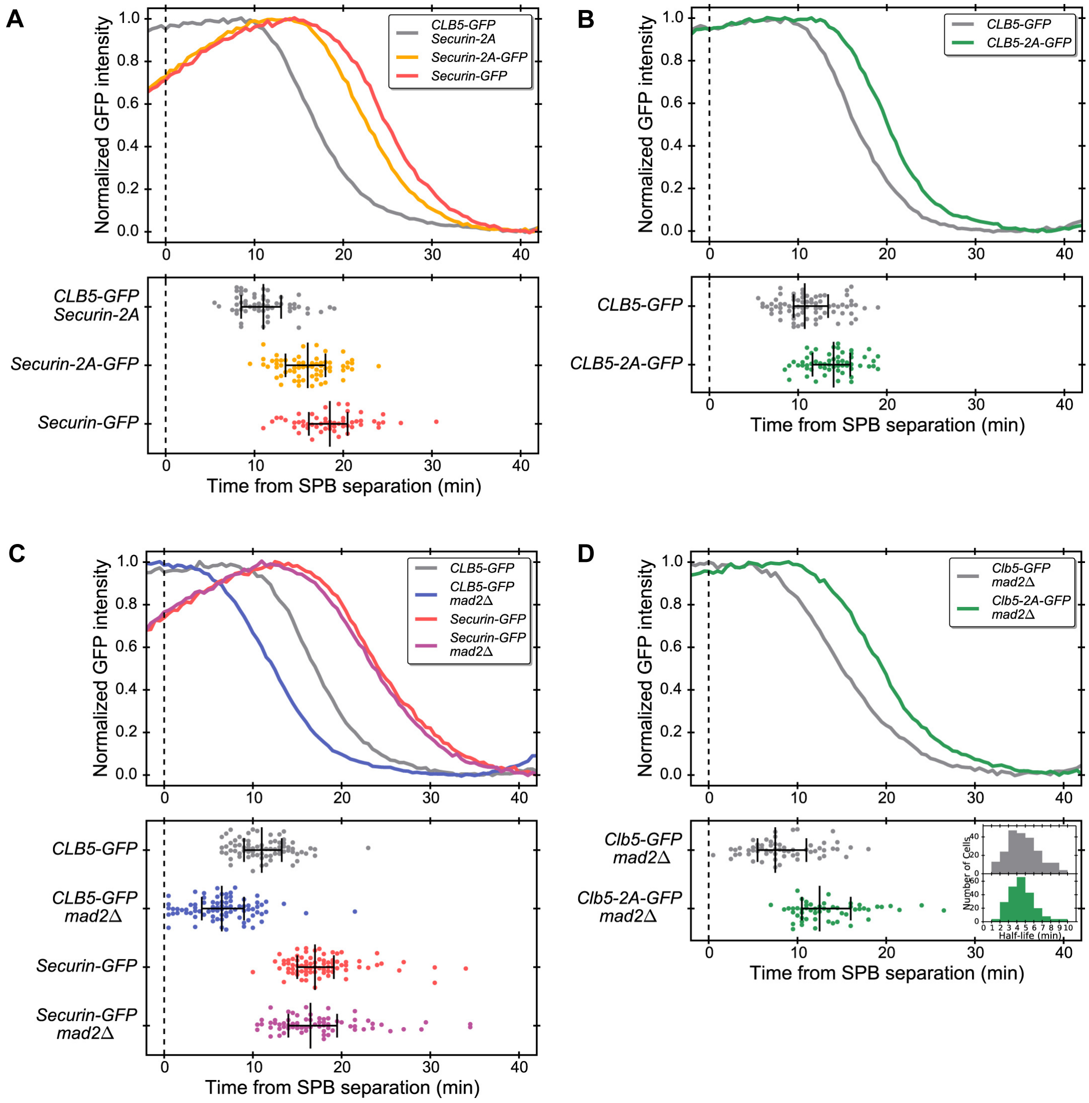

Fig. S3 Mechanisms that determine substrate degradation timing all change degradation onset. The method in Fig. S2 was used to determine the timing of APC/C^{Cdc20} substrate degradation onset in cells with different strain backgrounds, using our previously published data (Lu D, Hsiao JY, Davey NE, Van Voorhis VA, Foster SA, Tang C, Morgan DO. J Cell Biol. 2014;207:23-39). In the lower panels, each dot represents one cell, and the position of the dot along the x-axis indicates the time of degradation onset relative to the time of SPB separation (dashed line). Error bars indicate the 25th and 75th percentiles and the middle bar shows the median. N>50 for each strain. (A) Clb5 is degraded several minutes earlier than securin. Securin-2A has its Cdk1 phosphorylation sites mutated and is degraded earlier than wild-type securin. Clb5 degradation is identical either in a background with wild-type securin or securin-2A, and the plot shows Clb5 in securin-2A background. (B) Clb5-2A has its ABBA motif mutated and is degraded later than wild-type Clb5. (C) Cells lacking Mad2 have a disabled spindle assembly checkpoint and display earlier Clb5 degradation onset relative to wild-type cells. (D) Deletion of *MAD2* does not change the timing difference between Clb5 and Clb5-2A degradation onset. The insert in the lower right panel shows a histogram of rates of degradation, calculated from single cell traces.

$$\frac{dA}{dt} = p_a - k_a[S0][A] - k_a[S1][A] - k_a[S2][A] - k_a[S3][A] - k_a[S4][A] + k_d[AS0] + k_d[AS1] + k_d[AS2] + k_d[AS3] + k_d[AS4] + e[AS4] \quad (1)$$

$$\frac{dS0}{dt} = k_d[AS0] - k_a[A][S0] \quad (2)$$

$$\frac{dS1}{dt} = k_d[AS1] - k_a[A][S1] \quad (3)$$

$$\frac{dS2}{dt} = k_d[AS2] - k_a[A][S2] \quad (4)$$

$$\frac{dS3}{dt} = k_d[AS3] - k_a[A][S3] \quad (5)$$

$$\frac{dS4}{dt} = k_d[AS4] - k_a[A][S4] - e[S4] \quad (6)$$

$$\frac{dAS0}{dt} = -k_d[AS0] + k_a[S0][A] - k_{cat}[AS0] \quad (7)$$

$$\frac{dAS1}{dt} = k_{cat}[AS0] - k_d[AS1] + k_a[S1][A] - k_{cat}[AS1] \quad (8)$$

$$\frac{dAS2}{dt} = k_{cat}[AS1] - k_d[AS2] + k_a[S2][A] - k_{cat}[AS2] \quad (9)$$

$$\frac{dAS3}{dt} = k_{cat}[AS2] - k_d[AS3] + k_a[S3][A] - k_{cat}[AS3] \quad (10)$$

$$\frac{dAS4}{dt} = k_{cat}[AS3] - k_d[AS4] + k_a[S4][A] - e[AS4] \quad (11)$$

Fig. S4 Ordinary differential equations for analysis of substrate degradation in the one-substrate model.

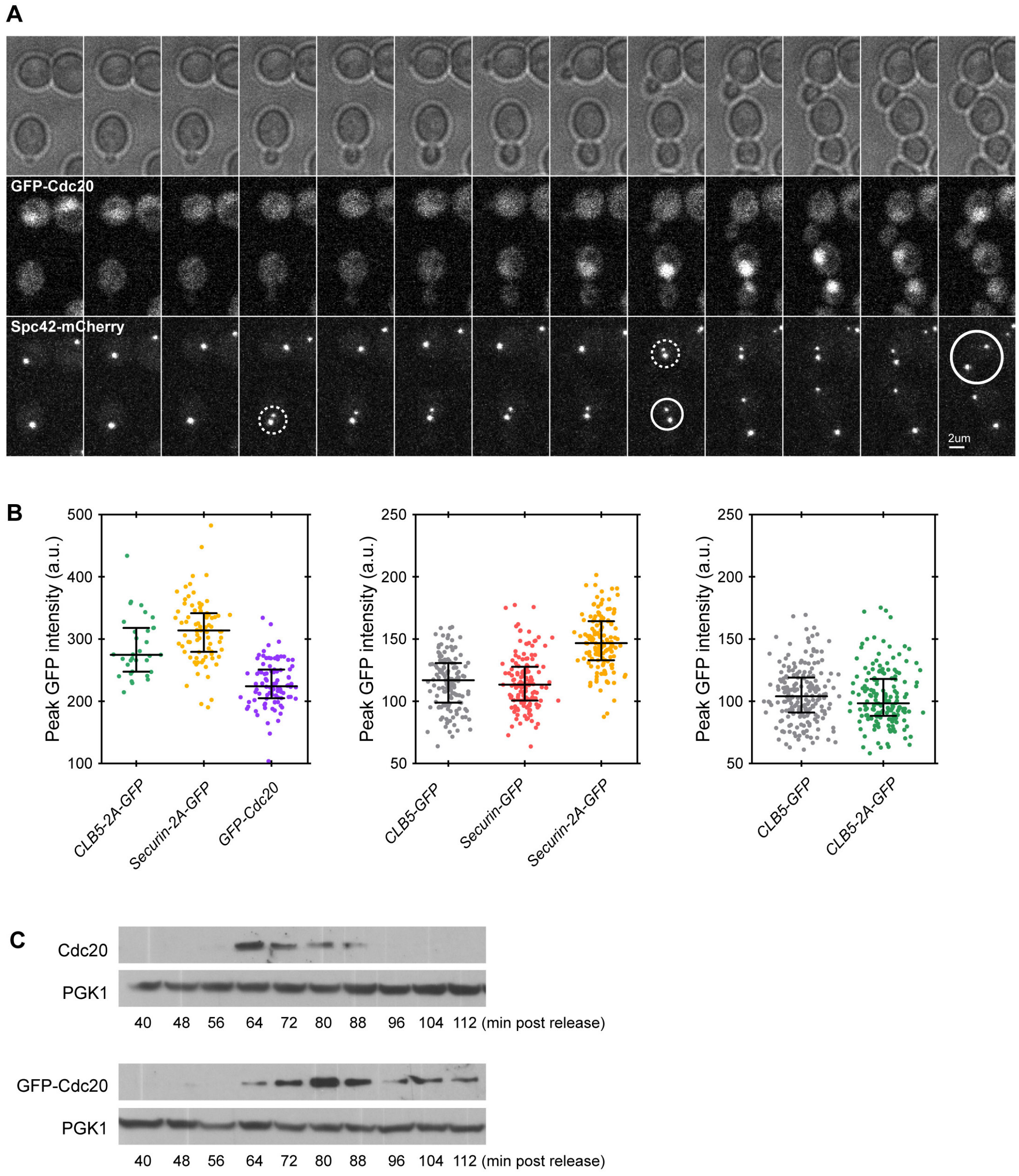

Fig. S5 APC/C^{Cdc20} levels in the cell are lower than those of its substrates. The amount of APC/C^{Cdc20} activity relative to substrates is a key determinant of substrate degradation dynamics. We reasoned that the amount of active APC/C^{Cdc20} should be no more than the amount of Cdc20 protein, so we sought to quantify the level of Cdc20 in the cell. We tagged endogenous Cdc20 with a fast-maturing GFP variant on its N-terminus to measure its concentration in single cells, relative to Clb5-GFP or securin-2A-GFP. (A) GFP-tagged Cdc20 and mCherry-tagged SPBs were analyzed in cycling cells at 4.5-min intervals. Dashed circles indicate cells at the onset of SPB separation, and solid circles mark cells at the onset of spindle elongation. (B) Left panel: peak GFP intensity was measured during a cell cycle in cells expressing Clb5-2A-GFP, securin-2A-GFP, or GFP-Cdc20. Middle and right panels: for comparison with

our previous experiments, previously published data (Lu D, Hsiao JY, Davey NE, Van Voorhis VA, Foster SA, Tang C, Morgan DO. J Cell Biol. 2014;207:23-39) were used to measure relative amounts of APC/C^{Cdc20} substrates using peak GFP levels during one cell cycle. Each dot is one cell. Error bars indicate the 25th and 75th percentiles and the middle bar shows the median. N>30 for each strain. (C) To control for the effect of GFP tagging on Cdc20 stability, we used western blotting with anti-Cdc20 antibodies to compare GFP-Cdc20 and untagged Cdc20. Cells with or without GFP-tagged Cdc20 were synchronized in G1 with alpha-factor and released. Samples were taken at the indicated time points and subjected to western blotting with anti-Cdc20 antibody. GFP tagging delayed Cdc20 protein accumulation and degradation, but peak levels of GFP-Cdc20 and untagged Cdc20 were comparable. Given that the peak levels of GFP-Cdc20 were slightly lower than those of Clb5-2A-GFP or securin-2A-GFP (panel B), we conclude that the concentration of Cdc20 in the cell is lower than that of its substrates.

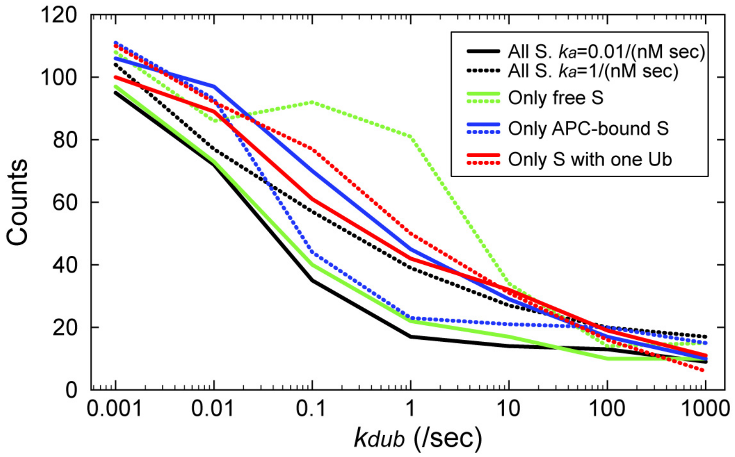

Fig. S6 Including deubiquitination in the model further limits the parameter space that generates a good delay in degradation onset and a fast rate of degradation. At k_a = 0.01/(nM sec) (solid lines) or k_a = 1/(nM sec) (dashed lines), the number of parameter combinations that meet the T95 and Td requirements was calculated as a function of increasing deubiquitination rate k_{dub}. Different colors correspond to different deubiquitination scenarios. Black: deubiquitination occurs for all substrates with ubiquitins attached, bound to APC/C^{Cdc20} or not; green: deubiquitination occurs only for free substrates; blue: deubiquitination occurs only for APC/C^{Cdc20}-bound substrates; red: deubiquitination occurs only for substrates with one ubiquitin attached.

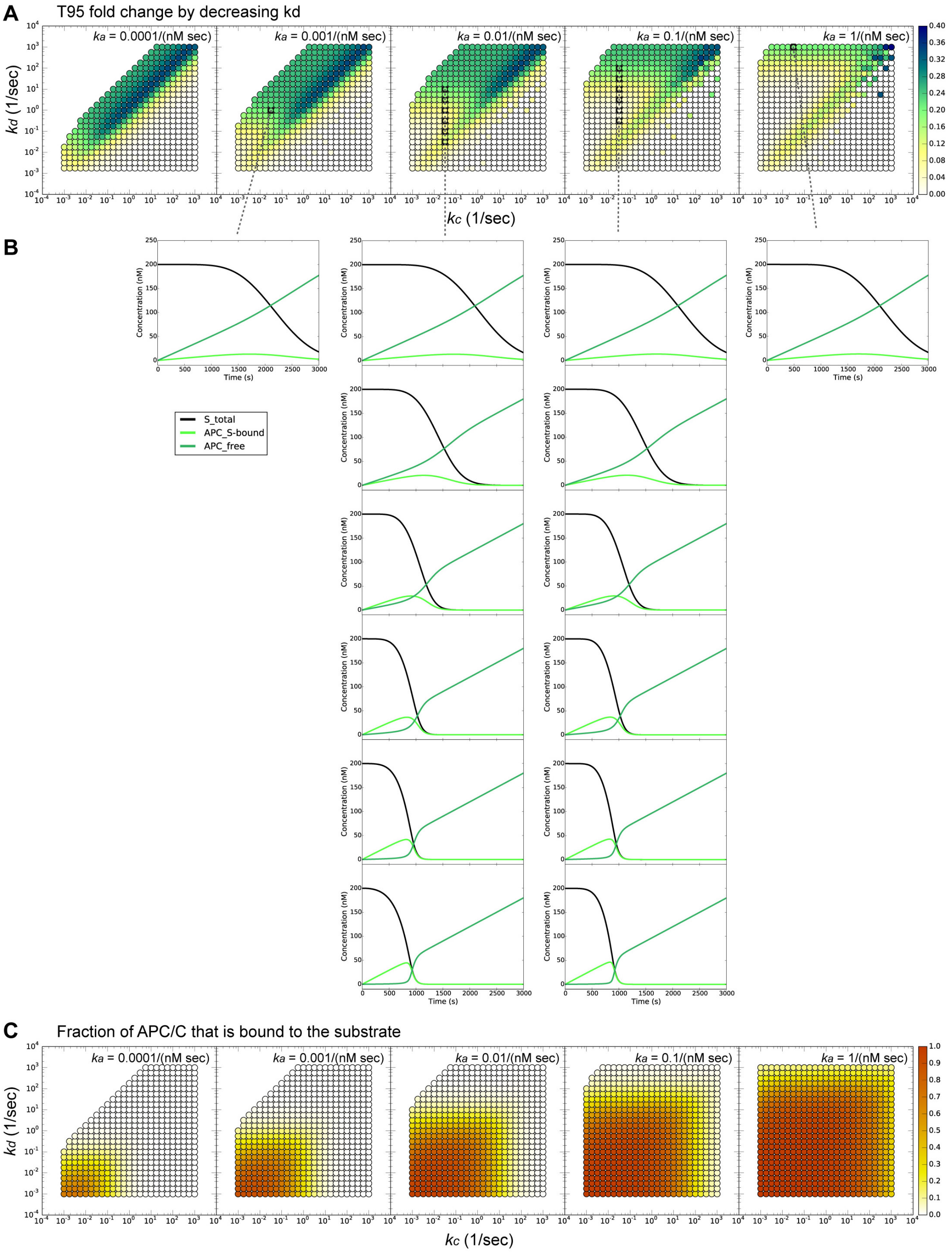

Fig. S7 Changing k_d influences T95 when not all APC/C^{Cdc20} is bound to the substrate. (A) Dot color indicates the relative change of T95 (as in Fig. 4B right panel) when k_d is decreased by a factor of 1.8, at different values of the association rate constant k_a. (B) Degradation profiles at different parameter combinations. Black curves show the total amount of substrate, light green curves show the amount of

substrate-bound APC/C^{Cdc20}, and dark green curves show the amount of free APC/C^{Cdc20}. From bottom to top, increasing k_d leads to significant increases in T95 only when there is enough free APC/C^{Cdc20}. On top row from left to right, for each plot, k_a and k_d increase simultaneously by 10 fold, and leads to similar degradation profiles. (C) The maximum fraction of substrate-bound APC/C^{Cdc20}, as in Fig. 4C.

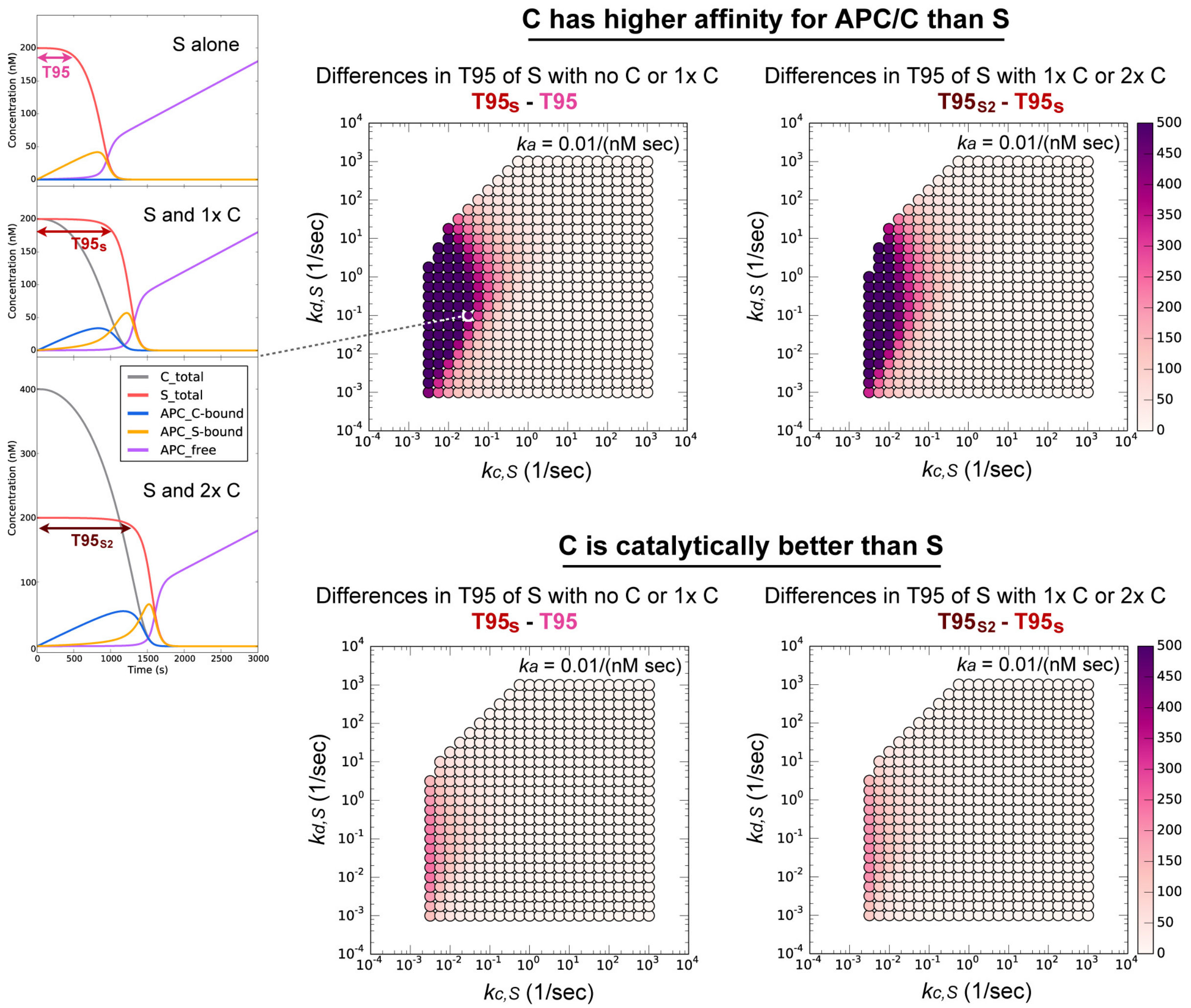

Fig. S8 Effects of doubling the concentration of C on T95 of S. These plots are generated using the same two-substrate models as in Fig. 5 and 6, where C either binds APC/C^{Cdc20} with 10-fold higher affinity than S (top panels) or C is catalytically 10-fold better than S (bottom panels). For each model scenario, the concentration of C is either 0 (equivalent to S being the only substrate, as in *clb5*Δ cells), the same as S (1x, as in wild-ype cells), or twice the concentration of S (2x, as in cells with two copies of *CLB5*). Comparing models with different amounts of C, the absolute differences in T95 of S are calculated across the same k_c-k_d parameter region as in previous figures. For comparison with our experimental results, these plots show the absolute values (in seconds) of T95 differences, rather than the relative T95 differences shown in Fig. 5C and 6C.
